# Leptin Enhances Synthesis of Proinflammatory Mediators in Human Osteoarthritic Cartilage—Mediator Role of NO in Leptin-Induced PGE_2_, IL-6, and IL-8 Production

**DOI:** 10.1155/2009/345838

**Published:** 2009-08-13

**Authors:** Katriina Vuolteenaho, Anna Koskinen, Meiju Kukkonen, Riina Nieminen, Unto Päivärinta, Teemu Moilanen, Eeva Moilanen

**Affiliations:** ^1^The Immunopharmacology Research Group, Medical School, University of Tampere and Research Unit, Tampere University Hospital, 33014 University of Tampere, Tampere, Finland; ^2^Coxa Hospital for Joint Replacement, P.O. Box 652, 33101 Tampere, Finland

## Abstract

Obesity is an important risk factor for osteoarthritis (OA) in weight-bearing joints, but also in hand joints, pointing to an obesity-related metabolic factor that influences on the pathogenesis of OA. Leptin is an adipokine regulating energy balance, and it has recently been related also to arthritis and inflammation as a proinflammatory factor. In the present paper, the effects of leptin on human OA cartilage were studied. Leptin alone or in combination with IL-1 enhanced the expression of iNOS and COX-2, and production of NO, PGE_2_, IL-6, and IL-8. The results suggest that the effects of leptin are mediated through activation of transcription factor nuclear factor *κ*B (NF-*κ*B) and mitogen-activated protein kinase (MAPK) pathway c-Jun NH_2_-terminal kinase (JNK). Interestingly, inhibition of leptin-induced NO production with a selective iNOS inhibitor 1400 W inhibited also the production of IL-6, IL-8, and PGE_2_, and this was reversed by exogenously added NO-donor SNAP, suggesting that the effects of leptin on IL-6, IL-8, and PGE_2_ production are dependent on NO. These findings support the idea of leptin as a factor enhancing the production of proinflammatory factors in OA cartilage and as an agent contributing to the obesity-associated increased risk for osteoarthritis.

## 1. Introduction

Leptin is a hormone that was initially found to be synthesized by white adipocytes and has a strong correlation with the amount of adipose tissue and with body mass index (BMI). Leptin was first discovered in 1994 and found to act as a signal for the central nervous system to inhibit food intake and to stimulate energy expenditure [1]. More recent findings on the ubiquitous expression of leptin receptors in almost all tissues and on its cellular effects have revealed that leptin is also involved in the regulation of a variety of biological functions related to immune responses and inflammatory diseases, and to cardiovascular and respiratory pathophysiology [2–5].

Obesity is an important risk factor for osteoarthritis (OA) [6]. Increased joint loading and altered mechanic loading axis has been proposed to explain the increased risk of OA in weight-bearing joints, including hip and knee joints. Surprisingly, there is also a positive association between obesity and OA in the hand, pointing to an obesity related metabolic factor that acts as a risk factor for OA. Recently, it has been shown that synovial fluid (SF) from OA patients contains leptin concentrations that are similar or higher than those measured in serum [7–9]. Furthermore, low soluble leptin receptor (sOb-R) level in SF potentiates the biological activity of leptin in the joint as compared to that in serum [8]. Articular cartilage has been reported to produce leptin [8, 10] and express functional leptin receptor Ob-R [11], and the expression of these two is further increased in advanced OA correlating with BMI of the patients [9].

In vitro, leptin has been shown to potentiate interleukin-1 (IL-1) and interferon *γ* (IFN*γ*)-induced production of nitric oxide (NO) in chondrocytes, which is a proinflammatory and destructive mediator in cartilage [12, 13]. Leptin has been shown to decrease chondrocyte proliferation and to increase production of proinflammatory cytokine IL-1*β* and destructive matrix metalloproteinases 9 and 13 (MMP-9 and 13) in human chondrocytes [9, 14]. On the other hand, leptin has also been reported to increase proliferation and to enhance proteoglycan and collagen synthesis in human chondrocytes [11]. In vivo, leptin injection into rat knee was reported to increase synthesis of insulin-like growth factor 1 (IGF-I) and transforming growth factor *β* (TGF*β*) both contributing to increased proteoglycan synthesis [7]. These effects are linked to increased cartilage matrix production, and also to osteophyte formation.

NO is related to the pathogenesis of OA as a destructive mediator [15]. Inducible nitric oxide synthase (iNOS) is expressed in OA cartilage, and there are markers of enhanced NO production in OA joints [15, 16]. Prostaglandins (PGs), especially PGE_2_, mediate inflammation, tissue destruction, and pain in OA and in OA joints they are formed by cyclooxygenase (COX) enzymes (particularly COX-2) and prostaglandin synthases [17]. Interleukin-6 (IL-6) and interleukin-8 (IL-8) are produced by OA cartilage and have a proinflammatory and modulatory role in the pathogenesis of OA [10, 18].

The presence of bioactive leptin in OA joint and the effects of leptin on cartilage metabolism point to a pathophysiological role for leptin in OA. The aim of the present study was to investigate the effects of leptin on mediators of cartilage metabolism by measuring its effects on the production of NO, PGE_2_, IL-6, and IL-8 in OA cartilage and by evaluating the signaling mechanisms involved in these effects by pharmacological means.

## 2. Materials and Methods

### 2.1. Patients and Cartilage Cultures

Cartilage tissue was obtained from the leftover pieces of total knee replacement surgery. The study was approved by the Ethics Committee of Tampere University Hospital, and the patients gave their written approval. The donor patients, age ranging from 53 to 87 years and body mass index ranging from 20 to 32, were all diagnosed to have osteoarthritis.

Cartilage samples were washed with phosphate buffered saline (PBS) and processed for the experiments within two hours after the operation. Full thickness pieces of articular cartilage from femoral condyles, tibial plateaus, and patellar surfaces showing macroscopical features of early OA were removed aseptically from subchondral bone with a scalpel and cut into small pieces (about 2 × 2 × 2 mm). Cartilage cubes randomly selected from 3 different areas of the joint were incubated in one well of a 6-well plate in 3 mL of tissue culture medium (Dulbecco's modified Eagle's medium (DMEM) with glutamax-I containing 10% heat-inactivated fetal bovine serum, penicillin (100 units/mL), streptomysin (100 *μ*g/mL), and amphotericin B (250 ng/mL); all obtained from Invitrogen, Carlsbad, Calif, USA)) at 37°C in humidified 5% carbon dioxide atmosphere.

In the first two series of experiments, OA cartilage explants from 8 patients were incubated with leptin (0.1 *μ*g/mL or 10 *μ*g/mL) alone or in combination with IL-1*β* (10 pg/mL) for 48 hours. Concentrations of NO, PGE_2_, IL-6, and IL-8 were determined in the culture medium. In the third series of experiments OA explants from 3 patients were incubated with leptin (0.1 *μ*g/mL or 10 *μ*g/mL) alone or in combination with IL-1*β* (10 pg/mL) for 48 hours. Cartilage samples were used to determine expression of iNOS and COX-2 protein. In the fourth series of experiments, signaling mechanisms involved in the leptin-induced NO, PGE_2_, IL-6, and IL-8 production were studied using pharmacological inhibitors. OA explants from 7 patients were incubated for 48 hours with leptin (10 *μ*g/mL) and following signaling pathway inhibitors: SP600125 10 *μ*M (JNK inhibitor), SB220025 0.5 *μ*M (p38 inhibitor), PD98059 10 *μ*M (Erk1/2 inhibitor), AG490 10 *μ*M (JAK2 inhibitor), WHI-P154 10 *μ*M (JAK3 inhibitor), Ro 31-8220 1 *μ*M (PKC inhibitor), MG132 10 *μ*M (NF-*κ*B inhibitor), and PDTC 10 *μ*M (NF-*κ*B inhibitor). Concentrations of NO, PGE_2_, IL-6, and IL-8 were determined in the culture medium. In the fifth series of experiments, the effect of NO on leptin-induced (10 *μ*g/mL) IL-6, IL-8, and PGE_2_ production was studied by inhibiting leptin-induced endogenous NO production with a selective iNOS inhibitor 1400 W (1 mM) during a 48 hour incubation of OA explants from 8 patients. The effect of NO was further investigated by studying if exogenous NO could reverse the effects of iNOS inhibitor 1400 W in leptin-treated cartilage. This was made by adding NO-donor SNAP (100 *μ*M) together with 1400 W and leptin in the cartilage cultures. Concentrations of NO, PGE_2_, IL-6, and IL-8 were determined in the culture medium.

After the experiment the cartilage explants were weighed, and the results were expressed per mg of cartilage. Aliquots of the culture media were kept at −20°C until assayed, and cartilage samples for Western blotting were first snap frozen in liquid nitrogen and analysed as described below.

### 2.2. NO Production

Concentrations of nitrite, a stable product of NO in aqueous solutions, were measured using the Griess reaction [19]. The results were expressed as pmol of nitrite/mg of cartilage.

### 2.3. Prostaglandin E_2_ Assays

The amount of PGE_2_ released into the incubation medium was determined by radioimmunoassay, using reagents from the Institute of Isotopes (Budapest, Hungary). The results were expressed as pg of PGE_2_/mg of cartilage.

### 2.4. IL-6 and IL-8 Assays

The concentrations of IL-6 (Sanquin, PeliPair, Amsterdam, The Netherlands) and IL-8 (R&D Systems, Minneapolis, MN, USA) in the culture medium were determined by ELISA. The results were expressed as pg of IL-6/mg of cartilage, or pg of IL-8/mg of cartilage.

### 2.5. Western Blot Analysis

After incubations, the cartilage specimen were snap frozen in liquid nitrogen, milled and lysed in extraction buffer (10 mM Tris-HCl, 5 mM EDTA, 50 mM NaCl, 1% Triton-X-100, 0.5 mM phenylmethylsulfonylfluoride (PMSF), 1 mM sodiumorthovanadate, 20 *μ*g/mL leupeptin, 50 *μ*g/mL aprotin, 5 mM sodium fluoride, 2 mM sodium pyrophosphate, 10 *μ*M n-octyl-beta-D-glucopyranoside). Following incubation on ice for 15 minutes, samples were centrifuged and supernatants were mixed with sample buffer 1 : 4 (62.5 mM Tris-HCl, pH 6.8, 10% glycerol, 2% SDS, 0.025% bromophenol blue, and 5% *β*-mercaptoethanol) and stored at −20°C until analyzed. Coomassie blue method was used to measure the protein content of the samples [20]. After boiling, protein samples (20 *μ*g) were separated with 8% SDS-polyacrylamide electrophoresis gels and transferred to Hybond enhanced chemiluminescence nitrocellulose membrane (Amersham Biosciences UK Limited, Buckinghamshire, UK). Proteins were identified by Western blotting using rabbit polyclonal antibody for human iNOS and goat polyclonal antibody for human COX-2 (both obtained from Santa Cruz Biotechnology, Santa Cruz, Calif, USA). Actin was analysed as a loading control by using rabbit polyclonal antibody (Santa Cruz Biotechnology, Santa Cruz, Calif, USA). Bound antibody was detected using SuperSignal West Pico chemiluminescent substrate (Pierce, Rockford, IL, USA) and FluorChem 8800 imaging system (Alpha Innotech, San Leandro, Calif, USA). Quantitation of the chemiluminescent signal was carried out with FluorChem software v.3.1.

### 2.6. Statistical Analysis

Results are expressed as mean ± standard error of the mean (SEM). Statistical significance of the results was calculated by using paired *t*-test.

### 2.7. Materials

Recombinant Human Leptin and Recombinant Human IL-1 beta were purchased from R&D Systems; manufacturer ensures low endotoxin level of the products (<1.0 EU per 1 *μ*g of the recombinant protein), and that amount in relation to the leptin concentrations used in the present study was tested to have no effect in our tissue culture conditions. Other reagents were obtained as follows: SP600125, SB220025, AG 490 and WHI-P154 were from Calbiochem (Merck, Darmstadt, Germany); PD 98059 was from Promega (Madison, WI, USA); Ro 31-8220 was from Alexis Corporation (Lausen, Switzerland); MG 132 was from Tocris Bioscience (Ellisville, MO, USA); PDTC was from Sigma Chemical Co (St Louis, MO, USA); SNAP was from Cayman Chemical (Ann Arbor, MI, USA). 1400 W was kindly given by Dr Richard G Knowles, Glaxo SmithKline Research & Development, Stevenage, UK.

## 3. Results

### 3.1. The Effects of Leptin on NO, PG*E*_2_, IL-6, and IL-8 Production in Human OA Cartilage

**Figure 1:**
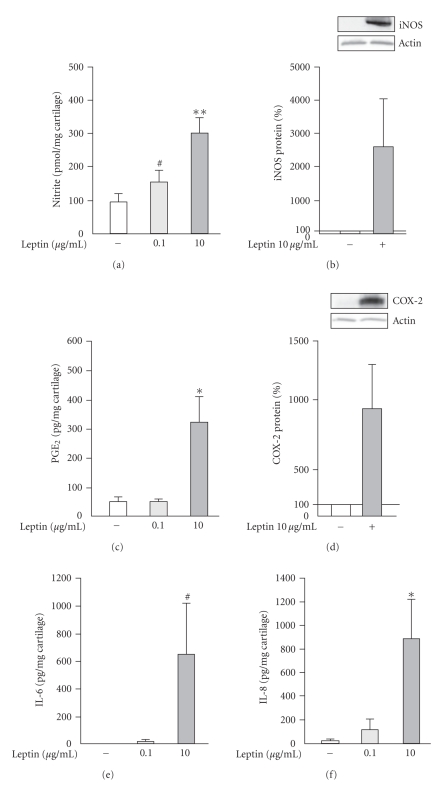
The effect of leptin on NO production (a), iNOS protein expression (b), PGE_2_ production (c), COX-2 protein expression (d), IL-6 production (e), and IL-8 production (f) in human OA cartilage. Cartilage explants were incubated with leptin (0.1 *μ*g/mL or 10 *μ*g/mL) for 48 hours. NO production (a) was measured as nitrite accumulation in the culture medium by Griess reaction. Expression of iNOS protein (b) and COX-2 protein (d) were measured by Western blot. PGE_2_ production (c) in the culture medium was measured by RIA. Concentrations of IL-6 (e), and IL-8 (f) in the culture medium were measured by ELISA. Results are expressed as pmol/mg cartilage (a), as percentages in comparison with control sample ((b) and (d)) or pg/mg cartilage ((c), (e), and (f)). Values are mean ± SEM. Samples were collected from 6 patients (*n* = 6) in (a) and (c), from 3 patients (*n* = 3) in (b) and (d), and from 7 patients (*n* = 7) in (e) and (f). *#*: *P* < .2, ∗: *P* < .05, and ∗ ∗: *P* < .01 as compared to control explants incubated in absence of exogenous leptin.

Leptin (0.1 *μ*g/mL and 10 *μ*g/mL) enhanced NO production in OA cartilage in a dose-dependent manner (Figure 1(a)). Western blot analysis with human iNOS antibody showed that leptin (10 *μ*g/mL) induced also iNOS expression in cultured cartilage tissue (Figure 1(b)). In addition, leptin (10 *μ*g/mL) increased PGE_2_ production and COX-2 expression, and IL-6 and IL-8 production in human OA cartilage during 48 hours incubation (Figures 1(c), 1(d), 1(e), and 1(f)).

**Figure 2:**
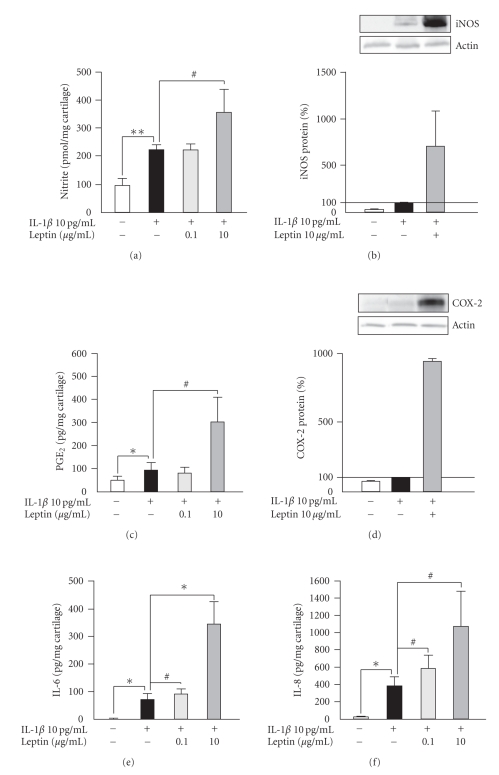
The effect of leptin on NO production (a), iNOS protein expression (b), PGE_2_ production (c), COX-2 protein expression (d), IL-6 production (e), and IL-8 production (f) in human OA cartilage in the presence of IL-1*β*. Cartilage explants were incubated with IL-1*β* (10 pg/mL) alone or in combination with leptin (0.1 *μ*g/mL or 10 *μ*g/mL) for 48 hours. NO production (a) was measured as nitrite accumulation in the culture medium by Griess reaction. Expression of iNOS protein (b) and COX-2 protein (d) were measured by Western blot. PGE_2_ production (c) in the culture medium was measured by RIA. Concentrations of IL-6 (e) and IL-8 (f) in the culture medium were measured by ELISA. Results are expressed as pmol/mg cartilage (a), as percentages in comparison with control sample ((b) and (d)) or pg/mg cartilage ((c), (e), and (f)). Values are mean ± SEM. Samples were collected from 6 patients (*n* = 6) in (a) and (c), from 3 patients (*n* = 3) in (b) and (d), and from 7 patients (*n* = 7) in (e) and (f). *#*: *P* < .2, ∗: *P* < .05, and ∗ ∗: *P* < .01.

A low concentration of proinflammatory cytokine IL-1*β* (10 pg/mL) had a slight stimulatory effect on NO, PGE_2_, IL-6, and IL-8 production and iNOS and COX-2 expression (Figure 2). Leptin (10 *μ*g/mL) enhanced NO, PGE_2_, IL-6, and IL-8 production, and iNOS and COX-2 expression in OA cartilage also in the presence of IL-1*β* (Figure 2).

### 3.2. Signaling Mechanisms Involved in the Leptin-Induced NO, PG*E*_2_, IL-6, and IL-8 Production

**Figure 3:**
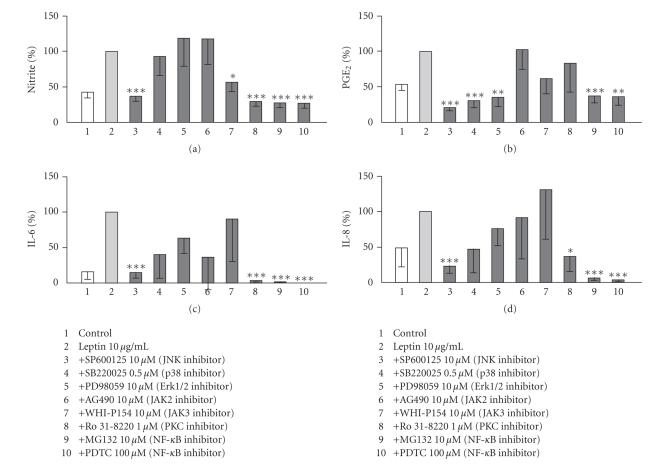
The effects of signaling pathway inhibitors on leptin induced NO (a), PGE_2_ (b), IL-6 (c), and IL-8 (d) production in human OA cartilage. Cartilage explants were incubated for 48 hours with leptin (10 *μ*g/mL) and the inhibitor indicated. NO production (a) was measured as nitrite accumulation in the culture medium by Griess reaction. PGE_2_ production (b) in the culture medium was measured by RIA. Levels of IL-6 (c), and IL-8 (d) in the culture medium were measured by ELISA. Leptin-induced NO/PGE_2_/IL-6/IL-8 production was set as 100%, and the other values were related to that. The results are expressed as mean ± SEM. Samples were collected from 7 patients in (a) and (b) (*n* = 7) and from 6 patients in (c) and (d) (*n* = 6). ∗: *P* < .05, ∗ ∗: *P* < .01, and ∗ ∗ ∗: *P* < .001 as compared to explants treated with leptin alone.

The involvement of signaling pathways (JNK, p38, and Erk1/2 MAP-kinases, JAK2 and JAK3, PKC, and transcription factor NF-*κ*B) in leptin-stimulated NO, PGE_2_, IL-6, and IL-8 production in OA cartilage was studied by pharmacological means. Inhibitors of transcription factor NF-*κ*B, MG 132 (10 *μ*M) and PDTC (100 *μ*M), and JNK inhibitor SP600125 (10 *μ*M) significantly inhibited leptin-induced NO, PGE_2_, IL-6, and IL-8 production (Figure 3). In addition to the effect of JNK inhibitor, inhibitors of other MAP-kinases, that is, SB220025 (p38 inhibitor; 0.5 *μ*M) and PD 98059 (Erk1/2 inhibitor; 10 *μ*M) inhibited leptin-induced PGE_2_ production, but had no effect on NO, IL-6, or IL-8 production. JAK2 inhibitor AG 490 (10 *μ*M) had no effect on leptin-induced NO, PGE_2_, IL-6, or IL-8 production, whereas JAK3 inhibitor WHI-P154 (10 *μ*M) inhibited leptin-induced NO synthesis, but not PGE_2_, IL-6, or IL-8 production. Leptin-induced NO, IL-6, and IL-8 production was inhibitable with protein kinase C inhibitor Ro 31-8220 (1 *μ*M), while it had no effect on PGE_2_ production.

### 3.3. The Effect of NO on Leptin-Induced IL-6, IL-8, and PG*E*_2_ Production

**Figure 4:**
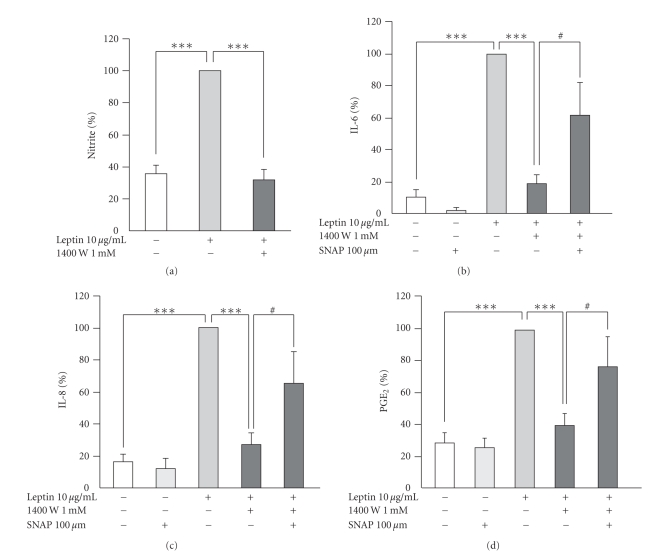
The effects of a selective iNOS inhibitor 1400 W (1 mM) ((a)–(d)) with and without NO-donor SNAP ((b)–(d)) on leptin (10 *μ*g/mL) induced NO (a), IL-6 (b), IL-8 (c), and PGE_2_ (d) production in OA cartilage during 48 hours incubation. In the culture medium, NO production (a) was measured as nitrite accumulation by Griess reaction, levels of IL-6 (b) and IL-8 (c) were measured by ELISA, and PGE_2_ production (d) was measured by RIA. Leptin-induced NO/PGE_2_/IL-6/IL-8 production was set as 100%, and the other values were related to that. The results are expressed as mean ± SEM. Samples were collected from 8 patients in (a) (*n* = 8), from 6 patients in (b) (*n* = 6), from 8 patients in (c) (*n* = 8), and from 6 patients in (d) (*n* = 6). *#*: *P* < .2, ∗ ∗ ∗: *P* < .001.

A selective iNOS inhibitor 1400 W (1 mM) inhibited leptin-induced NO production almost completely indicating that it was synthesized through iNOS pathway (Figure 4(a)). Interestingly, inhibition of NO production with 1400 W reduced also the production of IL-6, IL-8, and PGE_2_ (Figures 4(b)–4(d)). The effect of 1400 W was reversed when NO-donor SNAP (100 *μ*M) was added into the culture. Those results suggest that the increasing effect of leptin on IL-6, IL-8, and PGE_2_ production in human OA cartilage is dependent on NO.

## 4. Discussion

Osteoarthritis is a chronic disease characterised by gradual loss of the articular cartilage. The course of the destructive process is determined by the balance between anabolic and catabolic mediators and their regulators in the joint, and the local distribution of these mediators in the cartilage [18]. Leptin is an obesity related mediator, which has been suggested to take part in the regulation of anabolic and catabolic processes within the osteoarthritic joint and to play a role in the pathogenesis of OA [21]. In the present study, we found that leptin induced the production of NO, PGE_2_, IL-6, and IL-8 in human osteoarthritic cartilage and that leptin-induced PGE_2_, IL-6, and IL-8 production was dependent on NO. These findings support the role of leptin in the pathogenesis of OA.

NO mediates many of the destructive effects of IL-1 in inflamed joints [15, 16]. NO has been reported to increase the production of matrix metalloproteinases (MMPs) and to activate them [10, 22, 23], to inhibit proteoglycan [24–26] and collagen [27] synthesis and to induce chondrocyte cell death [28, 29]. NO is also involved in the progress of inflammation by reducing the production of anti-inflammatory/anabolic factors TGF-*β* [30], endogenous IL-1 receptor antagonist (IL-1ra), and IL-10 in chondrocytes [10, 31, 32], and by contributing to the resistance against anabolic effects of IGF-1 [33]. NO has also been shown to sustain activation of NF-*κ*B providing a prolonged transcription of NF-*κ*B dependent genes [34]. In support, Pelletier et al. reported reduced destruction of the articular cartilage by using iNOS-inhibitor L-NIL in instability-induced OA in dogs [35]. In further studies with this model, L-NIL was shown to reduce the levels of matrix metalloproteinase-1 and -3 (MMP-1 and -3) [23], to inhibit chondrocyte apoptosis [36] and to reduce the interleukin-1 converting enzyme (ICE) levels [37]. Van den Berg et al. studied the development of experimental osteoarthritis induced with intra-articular collagenase injection in iNOS knock-out mice. In this model, iNOS deficiency prevented the degree of cartilage destruction and osteophyte formation [38]. NO has several catabolic and antianabolic actions in cartilage and thus it is identified as a possible target of treatment in osteoarthritis.

Leptin has been shown to induce or to potentiate together with interferon *γ* (IFN*γ*), with tumor necrosis factor *α* (TNF*α*) or with IFN*γ* and IL-1*β*, NO production in murine J774A.1 macrophages, rat adipocytes, C6 glioma cell-line, and rat vascular smooth muscle cells (VSMCs) [39–42]. In human chondrocytes, leptin was reported to enhance interleukin-1 (IL-1) and interferon *γ* (IFN*γ*)-induced production of NO [12, 13]. In the present study, we showed that leptin alone is sufficient to induce the iNOS expression and NO production in human OA cartilage, and an enhancing effect was seen also in the presence of low concentrations of IL-1*β* (Figures 1(a), 1(b), and 2(a), 2(b)).

Prostaglandins (especially PGE_2_) are produced in high amounts in OA cartilage and are modulators of inflammation, tissue destruction, and inflammatory pain. Prostaglandins are formed from arachidonic acid by the prostaglandin synthesizing cyclooxygenase (COX) enzymes and prostaglandin synthases [17]. COX-2 is highly expressed in OA cartilage and is induced by various cytokines that are involved in destructive processes in OA cartilage, for example, IL-1 and TNF*α* [43–45]. Current drug therapy of OA is based on nonsteroidal anti-inflammatory drugs (NSAIDs). NSAIDs inhibit COX enzymes and prostanoid production, and they are used to relieve OA pain [46, 47]. To our knowledge, we show here for the first time that leptin increases COX-2 expression and PGE_2_ production in human OA cartilage in the absence and in the presence of IL-1 (Figures 1(c), 1(d) and 2(c), 2(d)). Previously, leptin has been shown to induce PGE_2_ production in OE33 Barret's esophageal adenocarcinoma (EAC) cell-line and in murine J774A.1 macrophages [39, 48].

Proinflammatory and regulatory cytokines IL-6 and IL-8 are found in SF from OA patients [49]. OA cartilage produces IL-6 and IL-8, and quantitative RT-PCR studies have shown elevated IL-6 and IL-8 mRNA levels in OA cartilage as compared to normal cartilage [10, 50]. Cytokines IL-1*β* and TNF*α* which induce destructive effects in cartilage both induce IL-6 and IL-8 production in human articular chondrocytes [51]. IL-6 and IL-8 may promote inflammation and cartilage destruction induced by IL-1 or TNF*α* and have a modulatory role in the pathogenesis of OA [18, 52–54]. In the present study, we show for the first time that leptin enhances IL-6 and IL-8 production in human OA cartilage (Figures 1(e), 1(f)  and 2(e), 2(f)). Leptin has previously been shown to induce IL-6 production in human dendritic cells, human monocytes, human endometrial stromal cells (ESC), and in epithelial cells [55–57], and to stimulate IL-8 production in human endometrial stromal cells (ESCs) and in epithelial cells [56].

Leptin signaling through leptin receptors (Ob-R) is thought to be mediated through janus kinase/signal transducer and activator of transcription (JAK/STAT) pathway and in addition to this, also mitogen-activated protein kinases (MAPKs), protein kinase C (PKC), phosphatidylinositol 3-kinase (PI3K), and nuclear factor *κ*B (NF-*κ*B) pathways have been reported to mediate some effects of leptin, depending on the cell type [3, 58]. In chondrocytes, Otero et al. showed by using pharmacological inhibitors that induction of NO production with a combination of leptin and IL-1 or IFN*γ* was dependent on JAK2, PI3K, Erk1/2, and p38 [12, 13, 59], but these studies did not show signaling pathways involved in the responses induced by leptin alone. We studied the effects of inhibitors of JAK (JAK2 and JAK3), MAPK (Erk1/2, p38, JNK), PKC and NF-*κ*B pathways on leptin-induced NO, PGE_2_, IL-6, and IL-8 production in OA cartilage. There seems to be some differences in the signaling pathways important for production of the four different leptin-induced inflammatory molecules in OA cartilage. Leptin-induced NO production was suppressed by inhibitors of JNK, JAK3, PKC, and NF-*κ*B, while leptin-induced PGE_2_ production was reduced by inhibitors of JNK, p38, and Erk1/2 MAPkinases and transcription factor NF-*κ*B. Leptin-induced IL-6 and IL-8 production was reduced by inhibitors of JNK, PKC, and NF-*κ*B (Figure 3).

In the present study, inhibition of leptin-induced endogenous NO production in OA cartilage with a selective iNOS inhibitor 1400 W also suppressed the effects of leptin on PGE_2_, IL-6, and IL-8 production. The effect was reversed with exogenously added NO (NO-donor SNAP) (Figure 4). Those results suggest that leptin induces PGE_2_, IL-6, and IL-8 production in OA cartilage by an NO-dependent manner. The mediator role of NO in leptin-induced metabolic changes in human OA cartilage has not been reported previously. It is, however, supported by the previous findings on the involvement of NO in the effects of leptin in some other organ systems, that is, in the secretion of luteinizing hormone-releasing hormone from the pituitary gland, in the control of blood pressure, and in the gastroprotection upon vagal activity [60–62].

In conclusion, OA cartilage was shown to respond to leptin by producing increased amounts of NO, PGE_2_, IL-6, IL-8, and all those effects can be considered harmful in cartilage metabolism. Those effects of leptin seem to be dependent on activation of transcription factor NF-*κ*B and the MAPK pathway c-Jun NH_2_-terminal kinase (JNK) in human OA cartilage. In addition, JAK3 signaling seems to be involved in leptin-induced NO production, p38, and Erk1/2 MAPK pathways in leptin-induced PGE_2_ production, and PKC pathway in leptin-induced NO, IL-6, and IL-8 production. Inhibition of NO production reduced the effects of leptin on PGE_2_, IL-6, and IL-8 production pointing to a mediator role of NO in these leptin-induced changes in cartilage metabolism and to a possible beneficial effect of iNOS inhibitors on OA cartilage. These findings support the idea of leptin as a factor in the pathogenesis of osteoarthritis, and as a possible link between obesity and osteoarthritis.
